# NGOs and government partnership for health systems strengthening: A qualitative study presenting viewpoints of government, NGOs and donors in Pakistan

**DOI:** 10.1186/1472-6963-11-122

**Published:** 2011-05-24

**Authors:** Iram Ejaz, Babar T Shaikh, Narjis Rizvi

**Affiliations:** 1 Health Systems Division, Department of Community Health Sciences, Aga Khan University Stadium Road, Karachi, Pakistan; 2 Health Services Academy, Opposite NIH, Chak Shahzad, Federal Ministry of Health, Islamabad, Pakistan

**Keywords:** non-governmental organizations, health systems strengthening, public private partnership, developing countries, Pakistan

## Abstract

**Background:**

Health systems are expected to serve the population needs in an effective, efficient and equitable manner. Therefore, the importance of strengthening of public, private and community health systems has been emphasized time and again. In most of the developing countries, certain weaknesses and gaps in the government health systems have been hampering the achievement of improved health outcomes. Public sector in Pakistan has been deficient in the capacity to deliver equitable and quality health services and thus has been grossly underutilized.

**Methods:**

A qualitative study comprising in-depth interviews was conducted capturing the perceptions of the government functionaries, NGO representatives and donor community about the role and position of NGOs in health systems strengthening in Pakistan's context. Analysis of the data was done manually to generate nodes, sub-nodes and themes.

**Results:**

Since many years, international and local non-governmental organizations (NGOs) have endeavored to fill the gaps in health service delivery, research and advocacy. NGOs have relatively performed better and achieved the results because of the flexible planning and the ability to design population based projects on health education, health promotion, social marketing, community development and advocacy. This paper captures the need and the opportunity of public private partnership in Pakistan and presents a framework for a meaningful engagement of the government and the private and nonprofit NGOs.

**Conclusion:**

Involving the NGOs for health system strengthening may eventually contribute to create a healthcare system reflecting an increased efficiency, more equity and good governance in the wake of the Millennium Development Goals. Nevertheless, few questions need to be answered and pre-requisites have to be fulfilled before moving on.

## Background

**Figure 1:**
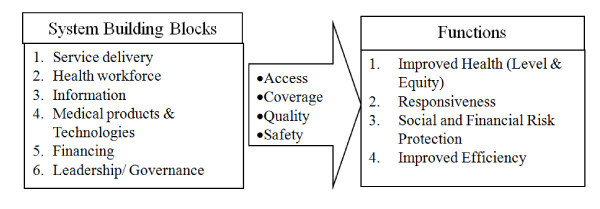
**The building blocks of the health systems: aims and attributes**. World Health Organization 2007.

The World Health Organization (WHO) defined health systems as "all the organizations, institutions, and resources that are devoted to producing health actions" in its World Health Report of the year 2000. This definition includes a full range of players engaged in the provision and financing of health services including public, nonprofit, and for-profit private sectors, as well as the international and bilateral donors, foundations, and the voluntary organizations involved in the funding or implementing health activities [1]. Health systems are expected to serve the population needs in an effective, efficient and equitable manner. Therefore, the World Health Organization later incorporated the efforts to influence determinants of health which made the health systems more than just the pyramid of publicly owned facilities that deliver personal health services [2]. Thus, the Health systems are at work at central, regional, district, community, and household levels, and hence all these entities need to be considered at all levels of discourse on health systems strengthening. The functions of health systems have been described most comprehensively in the World Health Report 2000 by WHO, and later elaborated in 2007 as shown in Figure 1.

Health system strengthening (HSS) is defined as any array of initiatives and strategies that improve one or more functions of the health system, leading to better health through improvements in the access, coverage, quality and safety [3]. Importance of strengthening of the public, private and community health systems has been emphasized in a variety of documents by various international, regional and national bodies concerned with the health care such as WHO, USAID, Global Fund etc. Weaknesses and gaps in the health systems limit the achievement of desired outcomes from the interventions at various levels and therefore impede the attainment of the broader national and international health care goals.

## Health sector in Pakistan

This has been evident now over a period of many years that the public sector in Pakistan is lacking in capacity in the context of delivery and management of health services [4,5]. Moreover, there are issues in the quality, efficiency and coverage of these services [6]. The dynamics of health planning in the history of Pakistan have been predominantly influenced by either the strong political agendas and manifestos or by the successive military regimens, marked by corruption and poor governance [7]. An independent report documented that the frequency of the informal payments to the public health care providers (which should be free of cost) amongst the users of services is 96% in Pakistan; most of these are demands from the providers at the health care facilities [8]. Political instability, lack of ownership of the programs by every next government and frequent transfers of the health care providers are further worsening the functioning of the public sector at large. The transfers of the health care providers are not need or merit based; rather these happen by virtue of the political influence and 'under the table payments' in most of the cases [9]. The healthcare system of Pakistan has always been inadequate and inept in meeting the needs of the ever growing populace [10]. Difficult or no access to health care services, extreme poverty, least awareness regarding maintenance of the health among the population, inadequate emphasis on addressing of the social determinants of health by the policy makers are some of the factors that worsen the situation of public health sector even more in the country. Some of the issues tend to exist for more than two decades now. One example pertains to the insufficient resources and their inefficient and ineffective use, leading to an inequitable provision of quality health care services. Another one relates to the discriminatory distribution of resources to government facilities in various provinces and regions of the country [11]. Due to the multitude of reasons, the primary health care is under-utilized and least productive in terms of its functions and achievement of its objectives [12]. Maintenance of the existing infrastructure and other resources within the public sector is another issue that has been influencing its functioning. Moreover, the public health sector has reflected the inability to cater the emerging needs for health care due to the population growth and the rising expectation on the quality of care [6]. Nonetheless, private sector has been a major contributor of the health care services in Pakistan. For financing, it is found that out of the total health expenditures in Pakistan, 33.6% is by the general government. The private expenditures constitute 64.5% of the total health expenditures in Pakistan, out of which 99.6% are the household's out of pocket health expenditures; most of which is spent on the purchase of drugs and payment of fee [13].

## Methods

This study had two pronged approach. Firstly, a literature search and a document review was carried out using the search engines like Google, Google Scholar™, Medline/PubMed and the Cochrane library. The key words used for this search were 'Health Systems Strengthening', 'Non-Governmental Organizations', 'Developing countries' and 'Public Private Partnership in Health Sector' and the combinations of these as well. Secondly, a qualitative exploratory research was conducted, using a semi-structured in-depth interview guide with the people holding key positions in the federal and provincial government (including program managers, technocrats, mid-level bureaucrats), non-governmental organizations (senior program managers or country directors) and the international donor community (country representatives or their deputies) at least. The NGOs included were those registered under the Societies Act of Pakistan and have been the recipient of donor's funding in the last 10 years. The final sample constituted of 5 participants from government, 8 from NGOs, and 5 from donor agencies. The in-depth interview guide was informed and developed with the help of the literature. The respondents were purposively selected, with at least one year of past experience of being involved in a public private venture in the health system of Pakistan. The era of partnerships focused for this study was 2000-2010. Verbatim notes of the in-depth interviews were transcribed to provide a record of what was said in the interviews. Tape recording was used where allowed by the study participants. The transcription and translation of data provided us with a descriptive record. The final interview notes were shared with the interviewees for validation. Another check of validity of transcriptions and the translations was done by a colleague who initially did not participate in the study. Analysis was carried out by manually whereby the process started during the data collection process and was thus well-connected to the final analysis. Coding or indexing was done for specific nodes and the probes of in-depth interviews keeping in view the interview guidelines. Key findings were aggregated and analyzed to develop the thematic areas.

Information gathered from the literature, interviews of various respondents from government ministries and programs, the representatives of NGOs and donors was then triangulated to find agreements and dissonances on variety of views and the issues around public private partnership. Further, the triangulation of themes arising from the interviews and literature review was done.

The ethical approval was obtained for this study and an informed consent for participating in the study was sought from each respondent. The study was conducted from January to September 2010.

## Results

The results will be presented as the findings of the literature and document review triangulated with the views of the study respondents captured from the qualitative study.

### 1. Recognition of importance and role of NGOs in health sector of Pakistan

Around 206 public private service organizations and 600 NGOs are engaged in health services provision, research and advocacy [14]. Since many years, the international and local NGOs have endeavored to fill the gaps that have been oft-cited for the public sector in Pakistan. These are mainly the lack of physical, financial, social and geographical access to the health care facilities, poor distribution of resources among various regions of the country, unavailability of health care providers at the facilities, poor quality of services at government health facilities and most importantly very little emphasis on addressing the social determinants of health due to weak inter-sectoral approach [15]. Working with NGOs besides offering financial benefits, represents a more attractive incentive which is the transfer of technical knowledge between partners. More so, health planning becomes far more participatory and consultative, with the inputs of all the stakeholders [16].

Most of the respondents from the government sector and the donor community emphasized the importance of NGOs at macro and micro levels of the health systems in Pakistan, giving a boost to the public sector while collaborating with various government departments. In the words of one of the government respondents:

*"In Pakistan, NGOs have always complemented the government's efforts and plugged in the gaps"*.

As per one donor representative,

*Over the time period government has realized that it can't do everything by itself. There has been more collaboration between the government and the NGOs during the late 1990s and early 2000s which involved term financing, pocket financing of NGOs, service delivery in number of different areas, e.g. HIV, TB, and now in management of basic health units"*. (Donor representative)

### 2. Outcomes of the past PPP projects

A remarkable escalation in the functioning of the NGOs has led to a large number of diverse projects and approaches. The NGOs have supplemented the government's efforts in monitoring the activities of many vertical programs [17]. There are already many successful public health programs implemented in collaboration with the NGOs including National Action Plan for the prevention and control of non-communicable diseases and health promotion (in collaboration with Heartfile, a non-governmental think tank) and the Leprosy Control Program (in collaboration with the Marie-Adelaide Leprosy Society) [6]. The tremendous success in these ventures is attributed to the fact that NGOs can promptly hire more staff (especially female health care providers) at acceptable salaries, acquire specialized equipment and execute ideal projects serving one limited population in a specified geographic area. Pakistan has benefitted substantially in the health sector through health education, health promotion, social marketing and advocacy by the not-for-profit private sector [18]. Though most of the NGOs have their presence in the urban and peri-urban areas; yet they have got great deal of strength for harmonizing with the larger public sector for health service delivery. A recent example of a successful public private partnership is the District Rahim Yar Khan Project whereby all the BHUs have been contracted-in to the Punjab Rural Support Program, an NGO. The broader aim of this project was to improve the primary health care services at the basic health units through better supervision of the staff and a functioning system of monitoring and regulation. The results are quite encouraging though precautions need to be taken for future scaling up of such initiatives [19]. In our study, more than half of the study participants talked about the success of this project particularly to improve the human resource management and make the health care providers available to the community at basic health facilities, i.e. Basic Health Units and Rural Health Centers.

*"Contracting of the PHC facilities has been tried out in many districts of Pakistan and has proved to be a huge success in terms of its outputs and the outcomes; however the plan for sustainability is still to be seen"*. (Government representative)

Many respondents recognized the contribution of NGOs in terms of their contribution towards the capacity building of human resource in the health sector across Pakistan.

*"Under the umbrella of national projects such as PAIMAN (The Pakistan Initiative for Mothers and Newborns), FALAH (Family Advancement for life and Health), and TACMIL (Technical Assistance for Capacity Building in Midwifery, Information and Logistics), numerous trainings have been imparted to various cadres of health personnel in order to strengthen the health care system of Pakistan"*. (NGO representative)

### 3. Role definitions of public and private partner within PPP

In a devolved system of government in Pakistan, the provincial and district health system have the opportunity to liaise closely with the private and the nonprofit sectors including the communities based organizations (CBOs), so as to best organize and coordinate PHC system [20]. Social health insurance or community based insurance, for instance, can be piloted through the NGOs who already have a rapport in the vulnerable communities. Similarly, involving the NGOs at governance level, for example, in the hospital management could help in achieving an efficiency and transparency in the public sector hospitals functioning. Private sector could also be involved in the accreditation and the continuing medical education of the medical practitioners [21]. Furthermore, it has been well established fact that another mode of improving the access and quality of service is social franchising whereby the private sector can be tapped for its potential [22]. There is a supportive environment for involving the NGOs and the CBOs in various other ventures, hence keeping the stewardship role with the government [23]. In addition to the clear demarcation of roles and responsibilities of each partner, setting of specified, realistic and shared objectives and maintenance of transparency, is another pre-requisite for a successful partnership.

When this aspect was inquired from the participants of the study, three main domains were mentioned as responses: "complement and supplement service delivery (ensuring quality, improving utilization, and through innovation); advocacy to influence policy; and capacity building of human resource".

*"NGOs, either local or international should have a role in terms of doing something innovative, something new to determine that this is what really works... ...They must have their role in bringing creativity; be it in training, advocacy or research"*. (NGO representative)

Moreover, NGOs are considered to be more effective in the community based health promotion activities.

*"Health promotion and health education is their art, because they are rooted into the societies by virtue of their work and because they enjoy a better rapport and trust of the community"*. (Government representative)

Participants were of the view that stewardship should be with the government; whereas the implementation should stay with the NGOs; and the monitoring of the activities should be done jointly.

### 4. Issues and challenges in PPP

Based on the current trend of government's engagement with the NGOs to strengthen the primary health care, certain challenges are highlighted in the literature; for instance, will it ensure equity, equality, efficiency in the service delivery and accountability in the system? How to best integrate the horizontal services with the vertical programs of the public sector, through interventions by the private and NGOs sector in order to maximize the output? What reforms must be envisaged to address the human resource issues and amongst them the managerial deficiencies through training and sensitization of the district level professionals in the public as well as the private sector? Could there be a consensus building among all stakeholders to ensure the sustainability of the projects especially those funded by the international donors [24]. Can strengthening of the district health system enhance the readiness and preparedness of the country to achieve the ambitious MDGs of reduction in child mortality, maternal mortality and an overall poverty in the country [25]?

In this regard, our study participants pointed out three main issues: lack of trust; capacity issues; and lack of clarity of roles and responsibility on both sides i.e. government and NGOs. Interestingly, a respondent from the government describes it as;

*"From conception of the idea till the monitoring and evaluation, there are opposite forces at all these levels; at the stage of MoU, there is resistance at ministerial levels, federal as well as provincial. At places, there is monopoly. We are yet to understand the usefulness of PPP. If government does its work properly, issues can be addressed"*. (Government representative).

At the same time, the dilemma that the government has to face when it wants to collaborate with the NGOs has been described by a representative of the donor agencies, who believes that:

*"If the government starts using NGOs instead of the normal public sector it is viewed as that the government does not trust its own system. So, I think until and unless the thinking changes at the strategic level and there is a clear policy push in that direction, the things won't change," *(Donor representative).

The NGOs and public sector keep putting responsibilities over each other to do certain things and basically it results from a lack of clarity in the roles and responsibilities of each sector while within partnership and also when working separately. Yet the respondents see a ray of hope.

*"Challenges are at every level; they are there to be faced and to be resolved. People who say that things can't be changed, and there is resistance from the government, they actually don't want to change. Obviously, if the government has not been able to do something previously and if an NGO wants to bring some change, then it's not easy against the status quo of the government. If you are persistent enough, only then you would be able to bring about a change." *(NGO representative)

### 5. Selection criteria and expectations from NGOs for a PPP venture

The NGO Code of Conduct for Health Systems Strengthening by World Health Organization (2009) is a fairly well written guide to help selecting the appropriate and capable NGOs [26]. It says:

I. NGOs will engage in hiring practices that ensure long-term health system sustainability.

II. NGOs will enact employee compensation practices that strengthen the public sector.

III. NGOs pledge to create and maintain human resources training and support systems that are good for the countries where they work.

IV. NGOs will minimize the NGO management burden for ministries.

V. NGOs will support Ministries of Health as they engage with communities.

VI. NGOs will advocate for policies that promote and support the public sector.

According to one government official,

*If these two sectors work in collaboration, they are capable enough to develop an enabling environment and synergy that will allow them to satisfy the needs of the people at large*. (Government representative)

However, the need for spelling out clear strategies for this engagement and for regulation of both partners has been emphasized time and again.

The participants very aptly pointed out few important points to be ascertained while devising the criterion for selecting the NGOs for partnership. According to one NGO representative who comprehensively covers everything, says that:

Following element should be accounted for: (i) *Structure: how many and how much resources are in command in the country; (ii) Space: how much social, cultural, ethical and economic space do the NGOs enjoy; (iii) Values: what kind of values are promoted by the NGOs; and (iv) Impact: what has been its impact in the past"*.

The donor perspective is slightly different which would entail what population has been served by that NGO in the past and how have they been doing in terms of quality, access and service provision.

## Discussion

### Need for a conceptual Framework for public private partnership

**Figure 2:**
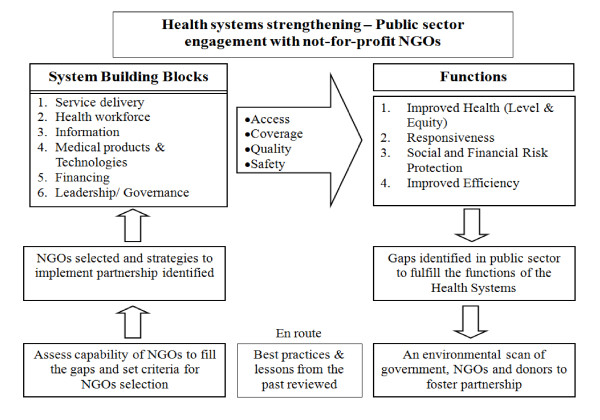
**Adapting WHO's framework for health systems strengthening through a meaningful public-private partnership**.

To achieve the targets set in the Millennium Development Goals (MDGs) by 2015, gross improvements in the quality and efficacy of medical care would require the strengthening of government's health programs and would surely necessitate collaborating with the NGOs [27]. The NGOs' engagement with public sector is to be seen as instrumental in attending to the issues of equity and quality improvement of the services provided along with dealing the issues of access and responsiveness of the system. For this meaningful collaboration, a framework would set the direction that will define mechanisms for the accountability and transparency. Nearly all of our study participants spoke about the gaps in the health sector that have become a *raison d'être *for NGOs to plug in with their expertise and scope of work. Expanding on the WHO's health systems building blocks, we present adapted framework which serves the prime objective of our study, as shown in Figure 2.

Gaps and flaws in the six building blocks of health systems can actually constrain achieving a universal access, coverage, quality and safety in the health care service delivery. NGO and private sector by virtue of its strengths and abilities has the potential of plugging these gaps in the public sector. Therefore, a partnership can eventually bear fruits of enhanced responsiveness, equity and efficiency in health care system. There are few questions, however, which would necessitate more in depth research on ground. Nevertheless, a careful analysis of all those factors which may or may not facilitate such partnership must be carried out in consultation with the key stakeholders, representing all relevant quarters in the government and the non-government entities.

## Conclusion

For any future public-private arrangements in health systems, it would be extremely desirable to carry out a mapping of the areas and sectors where government needs support from the NGOs. There on, it would be better to define clear roles and responsibilities of the parties; nature and timeline of deliverables and a clear plan of scaling up and sustainability. A meaningful public-private partnership may certainly help in fortifying the stewardship role of the government in terms of harnessing good governance and fostering more responsiveness in the health system of Pakistan.

## Competing interests

The authors declare that they have no competing interests.

## Authors' contributions

IE was involved in conception and design of the paper; analysis and interpretation of the literature; and drafting the paper; BTS contributed in revising it critically for substantial intellectual content and for adding references; and NR supervised the data collection and reviewed the write up. All authors read and approved the final manuscript.

## Pre-publication history

The pre-publication history for this paper can be accessed here:

http://www.biomedcentral.com/1472-6963/11/122/prepub
